# Facile synthesis of CsPbBr_3_/PbSe composite clusters

**DOI:** 10.1080/14686996.2017.1412231

**Published:** 2017-12-18

**Authors:** Thang Phan Nguyen, Abdullah Ozturk, Jongee Park, Woonbae Sohn, Tae Hyung Lee, Ho Won Jang, Soo Young Kim

**Affiliations:** ^a^ School of Chemical Engineering and Materials Science, Chung-Ang University, Seoul, Republic of Korea; ^b^ Metallurgical and Materials Engineering Department, Middle East Technical University, Ankara, Turkey; ^c^ Metallurgical and Materials Engineering Department, Atilim University, Ankara, Turkey; ^d^ Department of Materials Science and Engineering, Research Institute of Advanced Materials, Seoul National University, Seoul, Republic of Korea

**Keywords:** CsPbBr3, PbSe, cesium lead halide perovskite, nanocomposite, 20 Organic and soft materials (colloids, liquid crystals, gel, polymers), 102 Porous / Nanoporous / Nanostructured materials, 103 Composites

## Abstract

In this work, CsPbBr_3_ and PbSe nanocomposites were synthesized to protect perovskite material from self-enlargement during reaction. UV absorption and photoluminescence (PL) spectra indicate that the addition of Se into CsPbBr_3_ quantum dots modified the electronic structure of CsPbBr_3_, increasing the band gap from 2.38 to 2.48 eV as the Cs:Se ratio increased to 1:3. Thus, the emission color of CsPbBr_3_ perovskite quantum dots was modified from green to blue by increasing the Se ratio in composites. According to X-ray diffraction patterns, the structure of CsPbBr_3_ quantum dots changed from cubic to orthorhombic due to the introduction of PbSe at the surface. Transmission electron microscopy and X-ray photoemission spectroscopy confirmed that the atomic distribution in CsPbBr_3_/PbSe composite clusters is uniform and the composite materials were well formed. The PL intensity of a CsPbBr_3_/PbSe sample with a 1:1 Cs:Se ratio maintained 50% of its initial intensity after keeping the sample for 81 h in air, while the PL intensity of CsPbBr_3_ reduced to 20% of its initial intensity. Therefore, it is considered that low amounts of Se could improve the stability of CsPbBr_3_ quantum dots.

## Graphical abstract

**Figure:**
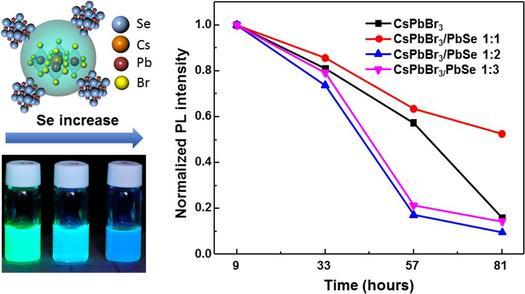

## 1. Introduction

Nowadays, much of the research concerning halide perovskites is focused on the new generation of solar cells, light-emitting diodes, transistors, lasers, and memristors with thin film, nanowire, and nanorod structures [1–6]. The power conversion efficiency of lead halide perovskite-based thin film photovoltaic cells has improved fast, from 3.8% to over 20% in a decade [7]. Among organic and inorganic perovskite materials, cesium lead halide has shown better stability than organic perovskite materials, suggesting great potential for use in opto-electronic devices.

Research on the morphology of perovskites shows that nano-size materials display superior properties in comparison with bulk-size materials, such as higher quantum yield, narrower emission bandwidth, and tunable color [8,9]. Recently, it was reported that cesium lead halide quantum dots have promisingly high color purity for light-emitting diodes [10,11]. Furthermore, it was shown that the growth rate, structure and size of CsPbBr_3_ dots can be controlled by changing the length of carbon chains of acids and amines in ligands during synthesis [12]. This allows the possibility for finding the optimal structure and size for cesium lead halide quantum dots. However, the thermal and electrical stability of cesium lead halide quantum dots is still limited.

One particular organo-halide perovskite material, CH_3_NH_3_PbI_3_, has been used as the shell of PbS quantum dots. It was reported that PbS/CH_3_NH_3_PbI_3_ core/shell quantum dots can increase the performance of sensitized solar cells because CH_3_NH_3_PbI_3_ covered the PbS core as a passive shell, improving the quantum efficiency and air stability [13]. Perovskite CH_3_NH_3_PbI_3_ was grown to cover the PbS quantum dots, which had been formed on a substrate. PbI_2_ was used as the Pb source of core PbS and the shell CH_3_NH_3_PbI_3_ layer. Nanocomposite synthesis with same base atom was reported in case of Nd_2_Sn_2_O_7_–SnO_2_, Dy_2_Sn_2_O_7_–SnO_2_, Nd_2_Zr_2_O_7_–ZrO_2_, and Ho_2_O_3_–SiO_2_ [14–21]. The recent reports about protecting perovskite materials were summarized in Table 1 [22–27].

**Table 1.** Recent methods to protect perovskite materials.

Perovskite	Additional materials	Structure	References
CsPbBr_3_	DBR mirror^a^	Protective layer	[22]
CsPbBr_3-x_I_x_	ZnS	Composite	[23]
FA-MAPbBr_3-x_I_x_^b^^,^^c^	Photopolymer	Protective layer	[24]
MAPbI_3_	C_6_H_5_(CH_2_)_2_NH_3_I	Layered hybrid structure	[25]
CsPbI_3_	bis-(2,2,4-trimethylpentyl)phosphinic acid (TMPPA)	Protecting solution for storing perovskite quantum dots	[26]
CsPbBr_3_/CsPbI_3_	NH_4_Br	Composite	[27]
CsPbBr_3_	PbSe	Composite	This work

^a^ DBR: Distributed Bragg reflector.

^b^ FA: Formamidinium.

^c^ MA: Methylammonium.

A nanocomposite material of CsPbBr_3_ and PbSe was synthesized to protect perovskite material from self-enlargement during reaction. As a core material, CsPbBr_3_ quantum dots were chosen because CsPbBr_3_ is the most stable among cesium lead halides. It is reported that materials with low lattice mismatch could easily form the core/shell structure such as CdSe/CdS (3.9% lattice mismatch) [28] or CdSe/ZnS (12% lattice mismatch) [29]. The lattice constant of cubic phase CsPbBr_3_ is known to be 5.87 Å and that of PbSe is reported to be 6.1 Å [30,31]. Therefore, the lattice mismatch between CsPbBr_3_ and PbSe is approximately 4%. The same source of Pb^2+^ in an organic solvent could be used to produce CsPbBr_3_ and PbSe. Furthermore, trioctylphosphine selenide can be used as an easy source for the PbSe shell layer. It is therefore expected that CsPbBr_3_/PbSe composites could be synthesized to improve the stability of inorganic perovskite quantum dots.

## 2. Experimental details

### 2.1. Materials

Lead bromide (PbBr_2_) 98%, cesium carbonate (Cs_2_CO_3_) 98%, octadecene (ODE) 90%, oleic acid 90% technique grade (OA), and oleyl amine (OAm) 95% were purchased from Sigma-Aldrich and used without further purification.

### 2.2. Synthesis of CsPbBr_3_ quantum dots

The synthesis of CsPbBr_3_ follows the previously reported procedures [32]. 70 mg of PbBr_2_ was loaded into a 100 mL flask along with 10 mL of ODE, 1 mL of OA, and 0.5 mL of OAm. The solution was then heated up to 120 °C under N_2_ to completely disperse PbBr_2_. Cesium oleate was prepared by loading 0.814 g of Cs_2_CO_3_ along with 30 mL of ODE and 2.5 mL of OA into a 100 mL flask. The temperature of the solution was increased to 120 °C for the completion of cesium oleate. After heating the PbBr_2_ solution to 190 °C, 0.4 mL of cesium oleate heated at 100 °C was injected to complete the synthesis of CsPbBr_3_ quantum dots. The mixture was cooled down in an ice water bath. Quantum dots were precipitated and washed using hexane and butanol with a 1:1 volume ratio. After that, quantum dots were dispersed into toluene for further use.

### 2.3. Synthesis of CsPbBr_3_/PbSe nanocomposite

The selenide source was prepared by loading 10 mg of selenium in 0.5 mL of trioctylphosphine (TOP) and 0.5 mL of ODE. The mixture was heated up to 140 °C in 1 h. The 0.2 mL of TOPSe was quickly added into the solution of PbBr_2_ with cesium oleate. The solution was kept at 190 °C for 1 h. Then, the color of the solution changed from light green to dark green. The materials were precipitated and washed using hexane and 2-butanol. The synthesized materials were re-dispersed in toluene for further use. In order to investigate the effect of synthesis temperature and the concentration of Se on the properties of the CsPbBr_3_/PbSe nanocomposite, samples were produced at synthesis temperatures of 150, 170, and 190 °C, and using 0.2, 0.4, and 0.6 mL of TOP-Se to match a Cs:Se mol ratio of 1:1, 1:2, and 1:3, respectively.

### 2.4. Characterization

A JASCO V-670 UV-vis spectrophotometer with a xenon arc lamp and PMT-1527 Hamamatsu photomultiplier was used to measure UV-visible absorbance and photoluminescence (PL) of the materials. X-ray diffraction (XRD, D8-Advance/Bruker-AXS), field-emission scanning electron microscopy (FE-SEM, SIGMA/Carl Zeiss), and transmission electron microscopy (TEM, JEOL-2100F, Japan) were applied to measure the structures and sizes of CsPbBr_3_ and CsPbBr_3_/PbSe composites. Synchrotron radiation photoemission spectroscopy (SRPES) experiments were also performed in an ultra-high-vacuum chamber (base pressure of ~10^−10^ Torr) at the 4D beam line of the Pohang Acceleration Laboratory. The onset of photoemission, corresponding to the vacuum level at the sample surface, was measured using an incident photon energy of 250 eV with a negative bias on the sample. The results were corrected for charging effects using Au 4f as an internal reference.

## 3. Results and discussion

**Figure 1.**
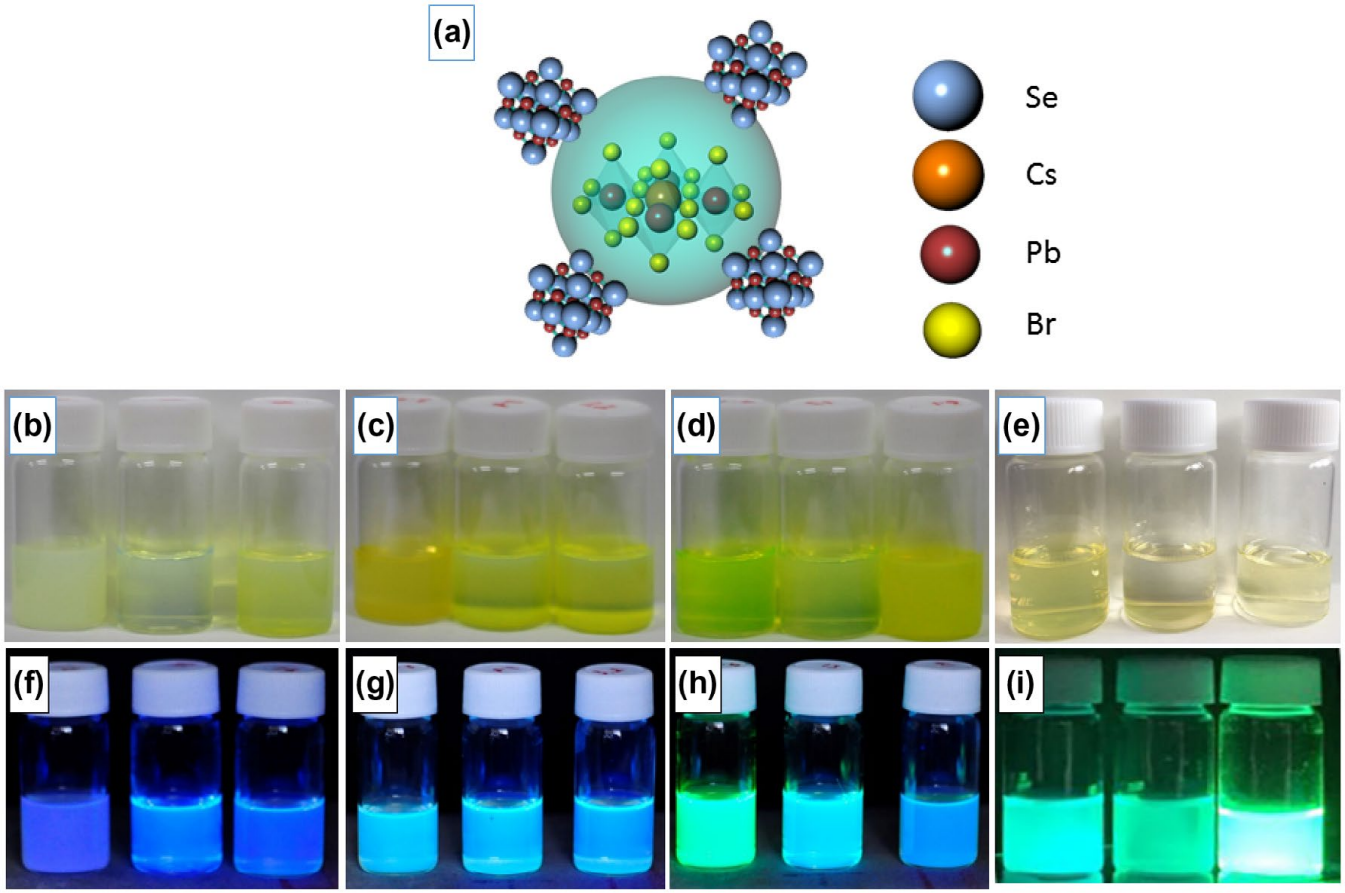
(a) Schematic of the expected CsPbBr_3_/PbSe nanocomposite structure. Photographs of CsPbBr_3_/PbSe nanocomposite in hexane solution under normal ((b)–(d)) and UV ((f)–(h)) illumination with Cs:Se ratios of 1:1, 1:2, and 1:3 synthesized at 150 °C ((b), (f)), 170 °C ((c), (g)) and 190 °C ((d), (h)) from left to right, respectively. For comparison, the photographs of pristine CsPbBr_3_ under (e) normal and (i) UV illumination are also shown.

The expected structure of CsPbBr_3_/PbSe composite is schematically drawn in Figure 1(a). Figure 1(b)–(i) show photographs of CsPbBr_3_/PbSe composites and pristine CsPbBr_3_ in hexane solution under normal light ((b)–(e)) and under a UV lamp ((f)–(i)). For comparison, the pristine CsPbBr_3_ is synthesized at 150, 170, and 190 °C from left to right as shown in Figure 1(e) and (i). The ratio of Cs:Se in photographs of CsPbBr_3_/PbSe composites is 1:1, 1:2, and 1:3 from left to right. The synthesis temperatures are 150 °C ((b), (f)), 170 °C ((c), (g)), and 190 °C ((d), (h)). The wavelength of the UV lamp was 365 nm. The colors of the composite solutions synthesized at 150 °C are all blue under UV illumination. At 170 °C, the solutions emitted light of cyan color under UV, suggesting that the light shifted to higher wavelength as the synthesis temperature increased. Composite materials synthesized at 190 °C emitted green light under UV illumination and the color of emitted light changed from green to cyan as the concentration of Se increased. These results indicate that the emission wavelength of CsPbBr_3_/PbSe composites under UV illumination increased as the synthesis temperature increased. Furthermore, the increase of Se concentration in CsPbBr_3_/PbSe shortened the wavelength of emitted light under UV illumination. The synthesis of perovskite quantum dots via solution methods is quite sensitive. A large size was simple to achieve due to the easy bonding of anion and cation. According to previous reports, the CsPbBr_3_ quantum dot solution was cooled down immediately after finishing the reaction [32,33]. In our case, the CsPbBr_3_ quantum dot solution was heated for 1 h after adding TOPSe to make CsPbBr_3_/PbSe composites. However, luminescence was detected under UV illumination as shown in Figure 1. This means that the crystal structure of CsPbBr_3_ was maintained even after a longer heating process and CsPbBr_3_ was protected by PbSe.

**Figure 2.**
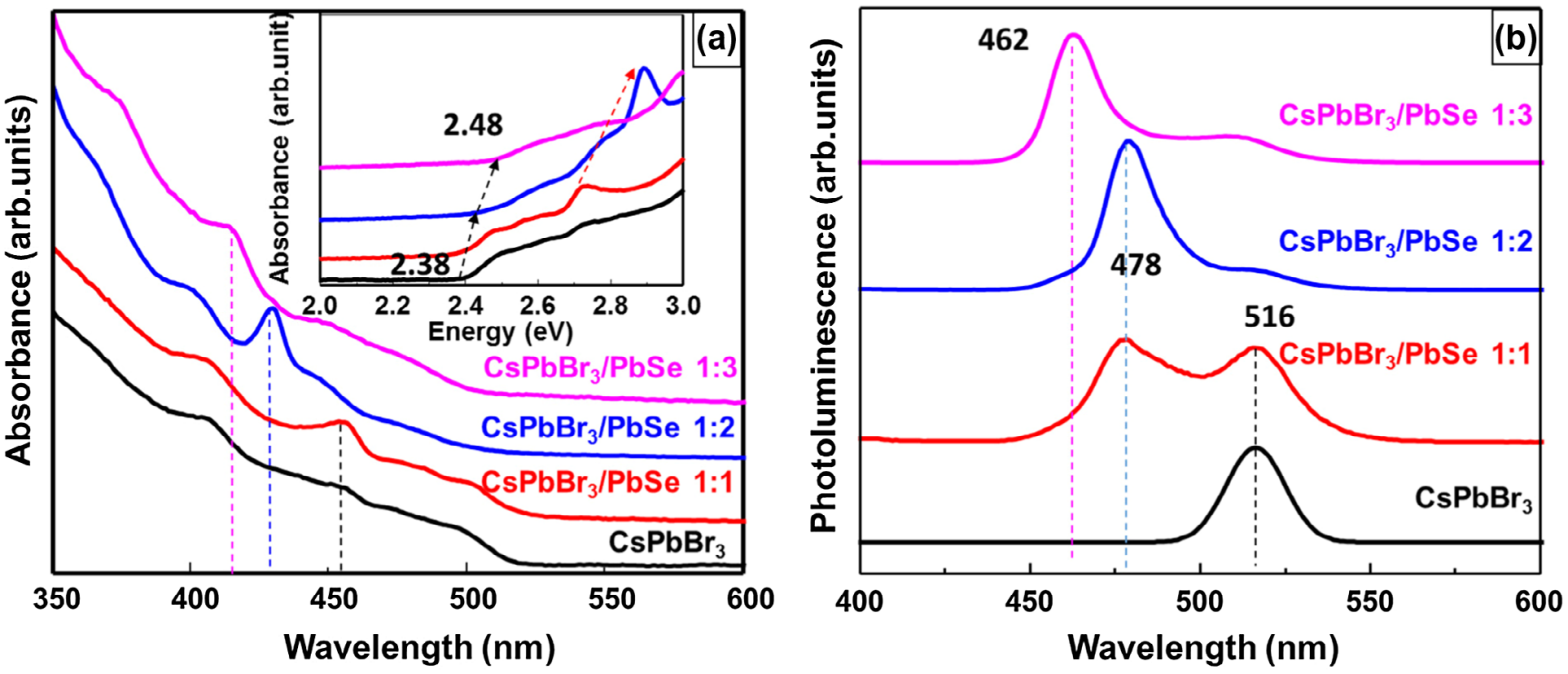
(a) UV-visible absorbance, and (b) photoluminescence spectra of bare CsPbBr_3_ quantum dots and CsPbBr_3_/PbSe nanocomposites. Spectra are vertically shifted for clarity.

To investigate the effects of the Se content of CsPbBr_3_/PbSe on its optical properties, UV-vis and PL spectra were measured. Figure 2 shows (a) UV-vis absorption spectra and (b) PL spectra of CsPbBr_3_ quantum dots and composites of CsPbBr_3_/PbSe with different Cs:Se ratios (1:1, 1:2, and 1:3). The synthesis temperature of the CsPbBr_3_/PbSe composites was fixed at 190 °C. The absorption peak appeared at 455 nm in CsPbBr_3_ quantum dots and in CsPbBr_3_/PbSe composites with a 1:1 Cs:Se ratio, which corresponds to an optical band gap of 2.38 eV as shown in the inset of Figure 2(a). In the case of CsPbBr_3_/PbSe composites with a 1:2 Cs:Se ratio, the absorption peak shifts to 429 nm, indicating that the band gap is increased to 2.42 eV. A Cs:Se ratio of 1:3 shifts the absorption peak to 414 nm, further increasing the band gap to 2.48 eV. These results indicate that the addition of Se into CsPbBr_3_ quantum dots modifies the electronic structure of CsPbBr_3_. This result is in agreement with the PL spectra as shown in Figure 2(b). The CsPbBr_3_ quantum dots emit green color with a wavelength between 500 and 550 nm. The PL spectra of CsPbBr_3_/PbSe composites with a 1:1 Cs:Se ratio indicate that their cyan color consists of a blue peak at 478 nm and a green peak at 516 nm. As the Cs:Se ratio increased from 1:1 to 1:3, the PL peak shifted to 462 nm. These results indicate that the emission color of CsPbBr_3_ perovskite quantum dots could be modified from green to blue by adjusting the Se ratio in composites.

**Figure 3.**
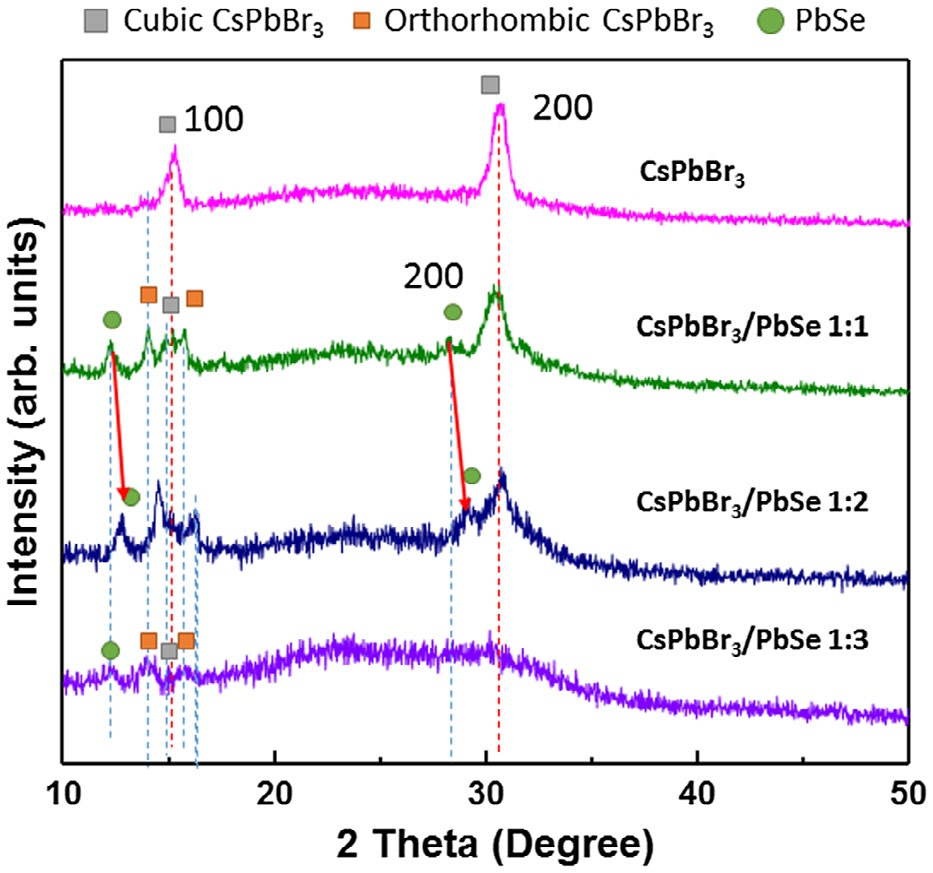
X-ray diffraction patterns of bare CsPbBr_3_ and CsPbBr_3_/PbSe nanocomposites with Cs:Se ratios of 1:1, 1:2, and 1:3 synthesized at 190 °C. The peaks of orthorhombic CsPbBr_3_ confirm the distortion of the lattice when forming PbSe beside the CsPbBr_3_ quantum dots in the 1:1 Cs:Se ratio sample.

In order to confirm the formation of PbSe on the surface of CsPbBr_3_ quantum dots, XRD measurements were performed. Figure 3 shows the XRD patterns of CsPbBr_3_ quantum dots and CsPbBr_3_/PbSe composites with different Cs:Se ratios (1:1, 1:2, and 1:3). The synthesis temperature of the CsPbBr_3_/PbSe composites was fixed at 190 °C. The CsPbBr_3_ quantum dots show two peaks at 15.16 and 30.6°, indicating that the synthesized CsPbBr_3_ quantum dots have a cubic structure with (1 0 0) and (2 0 0) planes. This result agrees well with previous reports on CsPbBr_3_ quantum dots synthesized at above 140 °C [32,34,35]. In the CsPbBr_3_/PbSe composite with a 1:1 Cs:Se ratio, new peaks appeared at 12.8 and 29.1° which correspond to the (1 0 0) and (2 0 0) planes of PbSe [36,37]. Furthermore, the (1 0 0) and (2 0 0) peaks of cubic CsPbBr_3_ disappeared and new peaks at 14 and 16° appeared, which are assigned to the (1 0 0) and (1 0 1) planes of orthorhombic CsPbBr_3_ [35]. This means that the structure of CsPbBr_3_ quantum dots changed from cubic to orthorhombic due to the introduction of PbSe at the surface. In the case of the CsPbBr_3_/PbSe composite with a 1:2 Cs:Se ratio, the peaks corresponding to PbSe (1 0 0) and (2 0 0) planes shifted to higher angles of 13 and 29°, respectively, suggesting that the plane distance of PbSe increased following the increase in Se. It is reported that PbSe has a cubic rocksalt structure with a lattice parameter of 6.12 Å and CsPbBr_3_ has perovskite structure with a lattice parameter of 5.87 Å [30,31]. Therefore, it is considered that PbSe was compressed due to the smaller lattice parameter of CsPbBr_3_ and that the lattice parameter of PbSe was restored as Se content increased. It is reported that orthorhombic phase has higher band gap than cubic phase. However, the photoluminescence is inactive in cubic-to-orthorhombic crystal transformation [26]. But, PL peak shift was found as shown in Figure 2. Therefore, band gap enlargement could be explained by Se doping. The (2 0 0) peak of CsPbBr_3_ was absent in XRD spectra. However, the (1 0 0) peak of PbSe and peaks of orthorhombic CsPbBr_3_ and (1 0 0) cubic CsPbBr_3_ appeared in XRD patterns with weak intensity. The full width at half maximum of PbSe and CsPbBr_3_ peaks also increased, indicating the size becomes smaller. Therefore, it is considered that the structure of cubic CsPbBr_3_ is broken as the ratio of Se increased. This result agrees with the data of absorbance and photoluminescence as shown in Figure 2.

**Figure 4.**
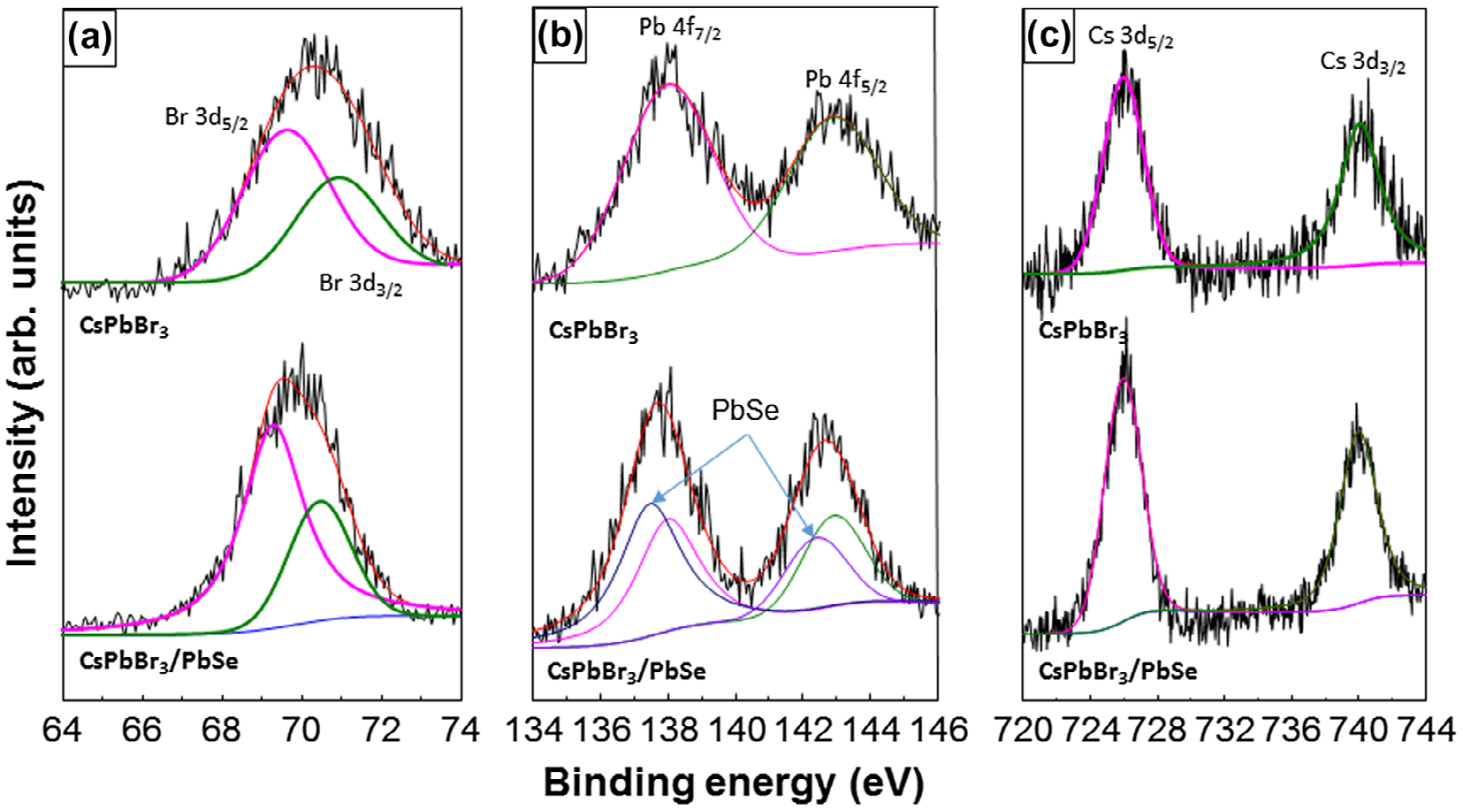
SRPES spectra of CsPbBr_3_ quantum dots and CsPbBr_3_/PbSe nanocomposites. The formation of PbSe peaks can be seen in the Pb 4f spectrum of the CsPbBr_3_/PbSe nanocomposites. The Br 3d and Cs 3d peaks do not show large changes, suggesting that the perovskite structure was unchanged by the PbSe growth.

Figure 4 shows SRPES spectra of CsPbBr_3_ quantum dots and CsPbBr_3_/PbSe composite synthesized at 190 °C with a Cs:Se ratio of 1:3. The two peaks of Cs 3d_5/2_ and Cs 3d_3/2_ are shown at 726.1 and 740 eV, respectively. The Br 3d_5/2_ and Br 3d_3/2_ peaks are also shown at 69.3 and 70.4 eV, respectively. No peak change was observed in Cs 3d and Br 3d after the formation of PbSe at the surface of CsPbBr_3_. In the case of the Pb 4f peak, two peaks are shown at 138.1 and 143 eV in CsPbBr_3_ quantum dots, which correspond to Pb 4f_7/2_ and Pb 4f_5/2_, respectively. Additional peaks appeared at 137.5 and 142.4 eV in the CsPbBr_3_/PbSe composite, which indicates the formation of Pb-Se bonds. These values are compatible with previous research on CsPbBr_3_ and PbSe nanocrystals [38–40]. These results indicate that PbSe was formed well on the surface of CsPbBr_3_ quantum dots.

**Figure 5.**
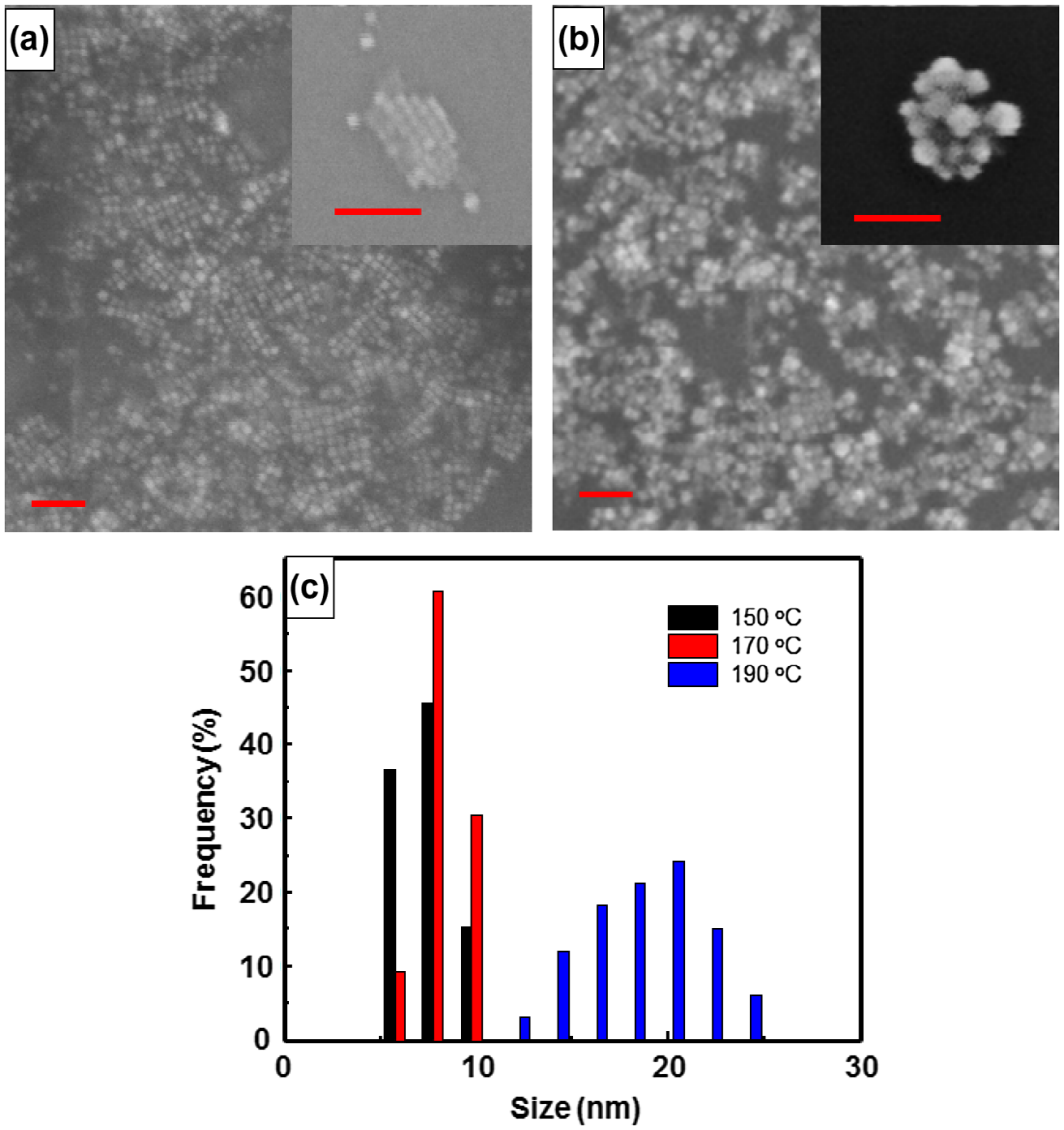
FE-SEM images of (a) CsPbBr_3_ quantum dots and (b) CsPbBr_3_/PbSe nanocomposite. Monodispersed CsPbBr_3_ quantum dots have a diameter of approximately 10 nm and nanocomposite clusters are approximately 20–23 nm in size. The scale bars are 100 nm. (c) Size distribution of CsPbBr_3_/PbSe synthesized at 150, 170, and 190 °C.

**Figure 6.**
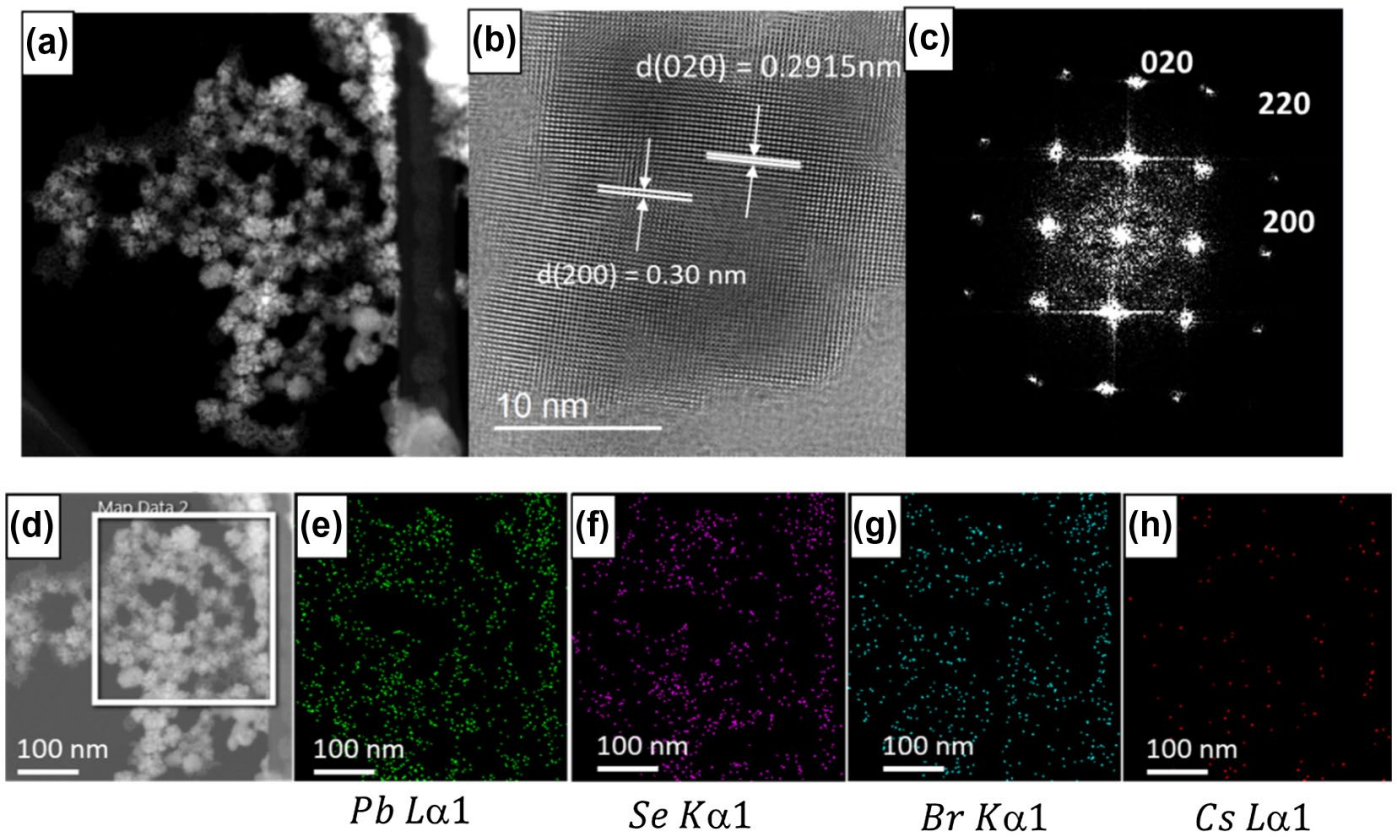
((a), (d)) TEM images of CsPbBr_3_/PbSe nanocomposite, (b) high-resolution TEM (HRTEM) image, (c) selected area electron diffraction (SAED) pattern, and EDS atomic mapping of (e) Pb, (f) Se, (g) Br, and (h) Cs. The HRTEM and SAED patterns show the (0 2 0) plane of CsPbBr_3_ and the (2 0 0) plane of PbSe with plane distances of approximately 0.3 nm.

The size of CsPbBr_3_ quantum dots and CsPbBr_3_/PbSe composite with a 1:3 Cs:Se ratio was measured using FE-SEM (Figure 5) and TEM (Figure 6). It is shown that the average size of CsPbBr_3_ quantum dots is approximately 15 nm and that of CsPbBr_3_/PbSe composite is approximately 20–25 nm. This suggests that PbSe is attached to the surface of CsPbBr_3_, increasing the size by approximately 5–8 nm. The magnified images shown in the insets of Figure 5(a) and (b) indicate that CsPbBr_3_ is mono-dispersed well and CsPbBr_3_/PbSe forms a cluster. Figure 5(c) shows the size distribution of CsPbBr_3_/PbSe composite clusters according to synthesis temperature. The average size of particles synthesized at 150, 170, and 190 °C is 6–8, 8–10, and 16–22 nm, respectively. These results support the conclusion that the emission wavelength of CsPbBr_3_/PbSe composites under UV illumination increased as the synthesis temperature increased (see Figures 1 and 2). The CsPbBr_3_/PbSe composite clusters were examined by TEM as shown in Figure 6. The (0 2 0) plane distance of CsPbBr_3_ was measured as 0.29 nm, and the (2 0 0) plane distance of PbSe had a similar value of 0.30 nm. Therefore, it is considered that the (0 2 0) plane of CsPbBr_3_ connects with the (2 0 0) plane of PbSe, forming the CsPbBr_3_/PbSe composite clusters. Energy dispersive X-ray spectroscopy (EDS) mapping of Pb, Se, Br, and Cs atoms confirmed that the atomic distribution in CsPbBr_3_/PbSe composite clusters is uniform and the composite materials were well-formed.

**Figure 7.**
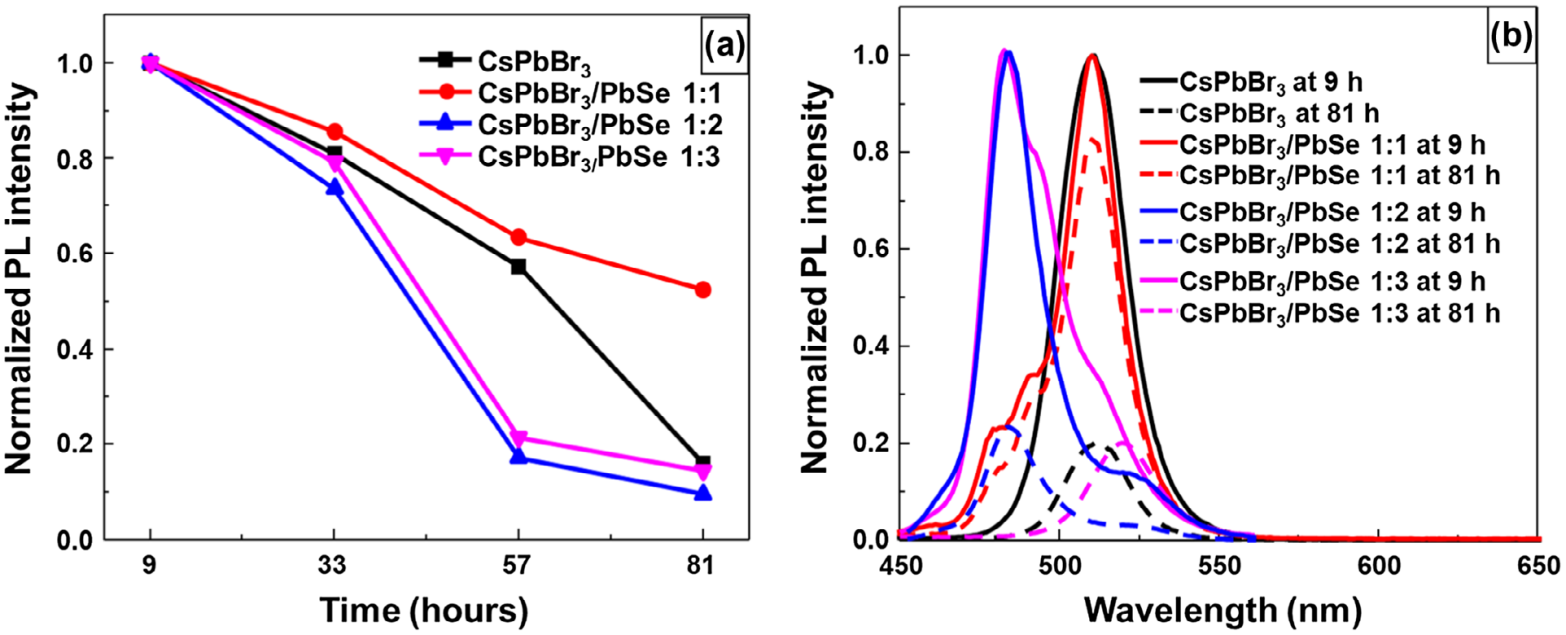
Stability of materials in hexane solution, stored under ambient air, as evaluated by photoluminescence measurements: (a) pristine and composite perovskites compared at 9, 33, 57, and 81 h; (b) normalized photoluminescence spectra of CsPbBr_3_ and CsPbBr_3_/PbSe 1:1, 1:2, 1:3 kept for 9 and 81 h.

In order to test their stability, CsPbBr_3_ quantum dots and CsPbBr_3_/PbSe composites synthesized at 190 °C were dispersed in hexane and kept in air. Figure 7(a) shows the change in normalized PL as a function of time. The PL was measured every 24 h from the starting point of measurement. After 33 h, the PL intensity had degraded to around 70–80% of its initial intensity regardless of samples. After 57 h, PL values of CsPbBr_3_/PbSe samples with Cs:Se ratios of 1:2 and 1:3 dramatically decreased to approximately 20% of its initial intensity, suggesting that PbSe could not elongate CsPbBr_3_ stability. In the case of CsPbBr_3_ quantum dots, the PL intensity reduced to 20% of its initial value after 81 h. However, CsPbBr_3_/PbSe with a 1:1 Cs:Se ratio still maintained 50% of its initial intensity even after 81 h, suggesting that low amounts of Se could improve the stability of CsPbBr_3_ quantum dots. Figure 7(b) shows normalized PL spectra after storage for 9 and 81 h. The reduction of photoluminescence after keeping samples in room condition was found even though the peak position was maintained. However, the shift peak to higher wavelength was found in CsPbBr_3_/PbSe composite synthesized at 190 °C with a Cs:Se ratio of 1:3. This result indicated that large amount of PbSe with CsPbBr_3_ could not protect perovskite quantum dots. We suppose the effect of PbSe with CsPbBr_3_ due to the stress of lattice mismatch. CsPbBr_3_ bonded due to the halide bonding that lead easily degrading in an ionic solvent or a humidity condition [32,41]. The high concentration of Se could cause the high stretch leading the degradation of CsPbBr_3_. With low concentration, the low stretch is not effect on CsPbBr_3_ and also protect the itself enlargement then improve the stability.

## 4. Conclusions

CsPbBr_3_/PbSe composite clusters were synthesized by adding TOPSe into a solution of PbBr_2_ with cesium oleate. As the ratio of Cs:Se increased from 1:1 to 1:3, CsPbBr_3_ partially transformed from cubic structure to orthorhombic structure and bonded with PbSe crystal, forming the composite cluster. The addition of Se into CsPbBr_3_ quantum dots modified the electronic structure of CsPbBr_3_, increasing the band gap from 2.38 to 2.48 eV as the Cs:Se ratio increased to 1:3. Therefore, the emission color of CsPbBr_3_ perovskite quantum dots was modified from green to blue by increasing the Se ratio in composites. The average size of CsPbBr_3_ quantum dots was approximately 15 nm and that of CsPbBr_3_/PbSe composite with a 1:3 Cs:Se ratio was approximately 20–23 nm. According to SEM and TEM images, CsPbBr_3_/PbSe composite exists in cluster form and each atom is uniformly distributed in the whole composite. The PL intensity of a CsPbBr_3_/PbSe sample with a 1:1 Cs:Se ratio maintained 50% of its initial intensity after keeping the sample for 81 h in air, while the PL intensity of CsPbBr_3_ quantum dots reduced to 20% of their initial intensity. These results suggested that low amounts of Se could improve the stability of CsPbBr_3_ quantum dots. Therefore, it is considered that this synthesis method for CsPbBr_3_/PbSe composites could be used for other perovskite materials to increase the stability.

## Disclosure statement

No potential conflict of interest was reported by the authors.

## Funding

This research was supported in part by a National Research Foundation of Korea (NRF) grant funded by the Korean government (MSIP) [grant number 2015K1A3A1A59073839] and in part by the Chung-Ang University Research Grants in 2016.
